# Performance of Meta’s Universal Model for Atoms across the Conformational and Configurational Space of Diverse Transition-Metal Catalysts

**DOI:** 10.1021/acs.jpca.5c07061

**Published:** 2026-02-18

**Authors:** Adarsh V. Kalikadien, Evgeny A. Pidko

**Affiliations:** Inorganic Systems Engineering, Department of Chemical Engineering, Faculty of Applied Sciences, 2860 Delft University of Technology, Van der Maasweg 9, 2629 HZ Delft, The Netherlands

## Abstract

Machine Learning Interatomic Potentials (MLIPs) promise to transform computational catalysis by delivering near-density functional theory (DFT) accuracy at a fraction of the computational cost. Here, we evaluate the Universal Machine Learning Potential for Atoms (UMA) on two data sets of transition-metal complexes. UMA enables high-throughput evaluations in seconds per structure on consumer-grade GPUs. Analysis of per-ligand Spearman rank correlations (ρ > 0.6, *p* < 0.05) reveals variability in ranking reliability that is not captured by aggregate metrics such as *R* ^2^ or RMSE. However, these inaccuracies are shown to mainly occur in the near-DFT accuracy regime where these complexes are practically indistinguishable. For square-planar Ni complexes, reliable rankings are obtained for 84% of ligands in rigid Ni–Cl_2_ complexes and drop to 53% for flexible asymmetric coordination environments, particularly only when conformers differ by <2 kJ/mol. Data set 2 shows a similar trend, with 61% and 44% reliability for Ru­(II) and Mn­(I) complexes, respectively, and, as expected, challenges for fluxional systems with small (<5 kJ/mol) relative energy gaps. These findings highlight the promise of MLIPs for both rigid, well-defined systems and highly flexible or fluxional catalysts, while underscoring the need to combine the speed of ML with validation and domain expertise to ensure robust and meaningful chemical insights.

## Graphical abstract

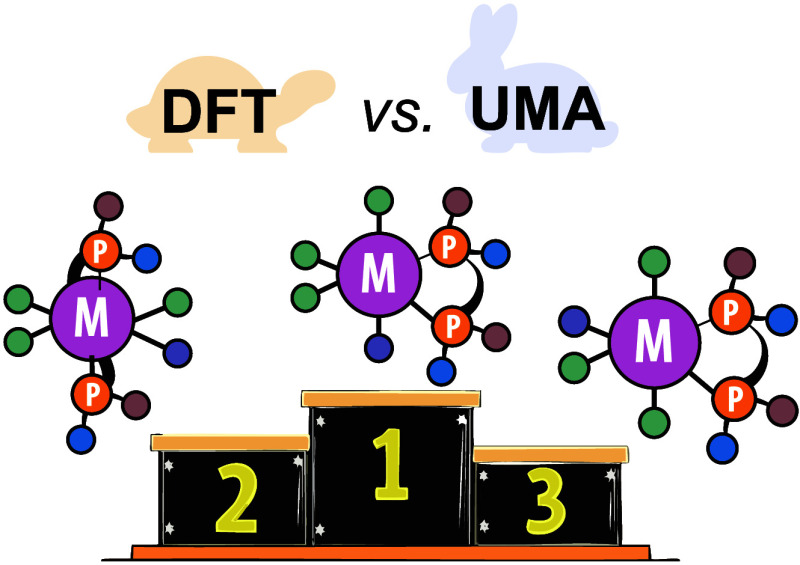

## 1 Introduction

Density functional theory (DFT) is indispensable for modeling catalytic reactions.^1–3^ In homogeneous catalysis, energetic barriers and molecular properties are typically derived from optimized geometries and their electronic structure.^1,4^ Although the molecular structures of these catalysts are relatively well-defined, their accurate calculations using even most modern quantum chemical methods come at a considerable computational cost.^5^ In fact, exhaustive computational sampling of the conformational and configurational space of a metal–ligand complex with DFT calculations can incur costs similar to the operating costs of wet-lab experimentation.

To illustrate this, consider a simple thought experiment. In high-performance computing (HPC), Standard Billing Units (SBUs) are used to quantify computational usage and cost, defined as the product of the number of CPU cores and the number of wall-time hours. In our experience, a typical TM complex requires approximately 4 h of computation using 32 CPU cores to optimize structure and carry out frequency analysis with hybrid DFT functional and double-ζ quality basis set. Given an optimistic estimate cost of €0.01 per SBU in The Netherlands, optimizing 10,000 such complexes would amount to roughly €12,800. In high-throughput screening efforts, such scales are common.^6,7^ It is, therefore, essential to develop reductionist approaches that maintain chemical accuracy while significantly lowering computational demand.^6,8^

Machine Learning Interatomic Potentials (MLIPs) offer a promising solution, enabling the approximation of DFT-level energies within seconds.^9–12^ In the early seminal papers, MLIPs were limited to generating interatomic potentials for highly specific systems.^12,13^ Unfortunately, to this day, a central challenge in developing MLIPs still lies in achieving sufficient generalization across the diverse domains and tasks for which DFT is employed.^14–16^ Recently, Meta released large, chemically diverse data sets designed to support general-purpose models.^17,18^ Alongside these data sets, a family of Universal Models for Atoms (UMA) was presented.^18^ These general-purpose models have demonstrated competitive or superior performance in terms of accuracy, inference speed, and memory efficiency when benchmarked against specialized models across a wide range of molecules.^18^ An exciting feature of UMA is that automated workflows were used to include an extremely large number of TM complexes in the Open Molecules 2025 (OMOL25) data set, allowing for extensive sampling of conformer space and configurational flexibility across different DFT data sets and tasks.^18^ However, the ranking of conformers has only been evaluated for a subset of the data set (GEOM) aimed at drug-like molecules.^18,19^

An ideal long-term goal would be to employ MLIPs directly in geometry optimization, where both energies and forces are evaluated by the MLIP, bypassing the need for DFT calculations. However, while active research is being devoted to this area, the methodology is not yet stable enough for routine application to complex systems.^20,21^ In the present work, therefore, we restrict our focus to assessing how well UMA can reproduce DFT-calculated energies on DFT-optimized geometries for catalytically relevant organometallic complexes with a particular focus on the correct description of conformational and configurational ensembles.

Specifically, we investigate the ability of UMA to rank the relative stability of TM complexes with varying conformational and configurational flexibility. Given the high inference speed of UMA, we assessed whether the predicted relative stabilities of different configurations of diverse transition-metal complexes are accurate enough for practical use cases. To address this question, we evaluate the performance of the smallest UMA model using our previously published data sets containing DFT-optimized geometries and energies for a broad array of TM complexes with varying bisphosphine ligands.

## 2 Computational Methods

In this study, the latest version of the small UMA model (UMA-s-1.1) pretrained on the OMol25 data set with 150 M total parameters was used. This model was chosen because we were mainly interested in a single-point energy calculation of structures with less than 1k atoms. Single-point energy calculations were performed via the Atomic Simulation Environment (ASE) in Python on a Dell X-ray photoelectron spectroscopy (XPS) 15 9520 laptop with a 12th generation Intel Core i5 processor, 32GB of RAM, and a Nvidia RTX 3050 GPU.

All generated DFT data sets focus on transition-metal based catalysts with bisphosphine ligands (Figure 1). Data set 1 consists of conformers for square-planar Ni-based catalyst structures with 25 varying bisphosphine ligands. Two surrogate “model” structures were used to represent the catalyst, each exhibiting different degrees of conformational flexibility: a rigid [ligand]-Ni­(II)-Cl_2_ complex (top left), in which Cl_2_ creates a symmetric coordination environment, and a more flexible [ligand]-Ni­(II)-(CH_3_CN)­(−pOMe­(C_6_H_4_)) complex (top right), where asymmetry is introduced by the coordination environment which increases conformational freedom.^23^ These complexes are viewed as representative models of the precatalysts and relevant intermediate in the Ni-catalyzed arylation of nitriles. Data set 2 contains various octahedral complexes with Ir, Ru, or Mn metal centers in combination with 88 bisphosphine ligands relevant for homogeneous hydrogenation catalysis presented in our previous research.^22^ The studied ligand configurations (bottom) for the Ir, Ru, and Mn complexes are named according to the donor atoms of ligands in the axial position.^22^ For both data sets, the atomic structures of the coordination complexes including all possible stereoisomers were generated with our MACE Python package,^24^ followed by exhaustive conformer search. For Data set 1, this conformer search was performed with CREST at the GFN2-xTB level of theory,^25–27^ after which all resulting structures were subjected to density functional theory calculations. For Data set 2, conformers were generated using RDkit^28^ and the lowest-energy conformer per ligand was selected for further analysis. Density functional theory calculations in gas phase were then used to optimize all of the resulting structures at the PBE0-D3­(BJ)/def2-SVP level of theory.^29–32^ Normal mode analysis was carried out to confirm that the optimized geometries correspond to local minima on the potential energy surface. For structures with imaginary frequencies, the PyQRC python package was used to remove these imaginary frequencies and restart geometry optimizations.^33,34^

**Figure 1:**
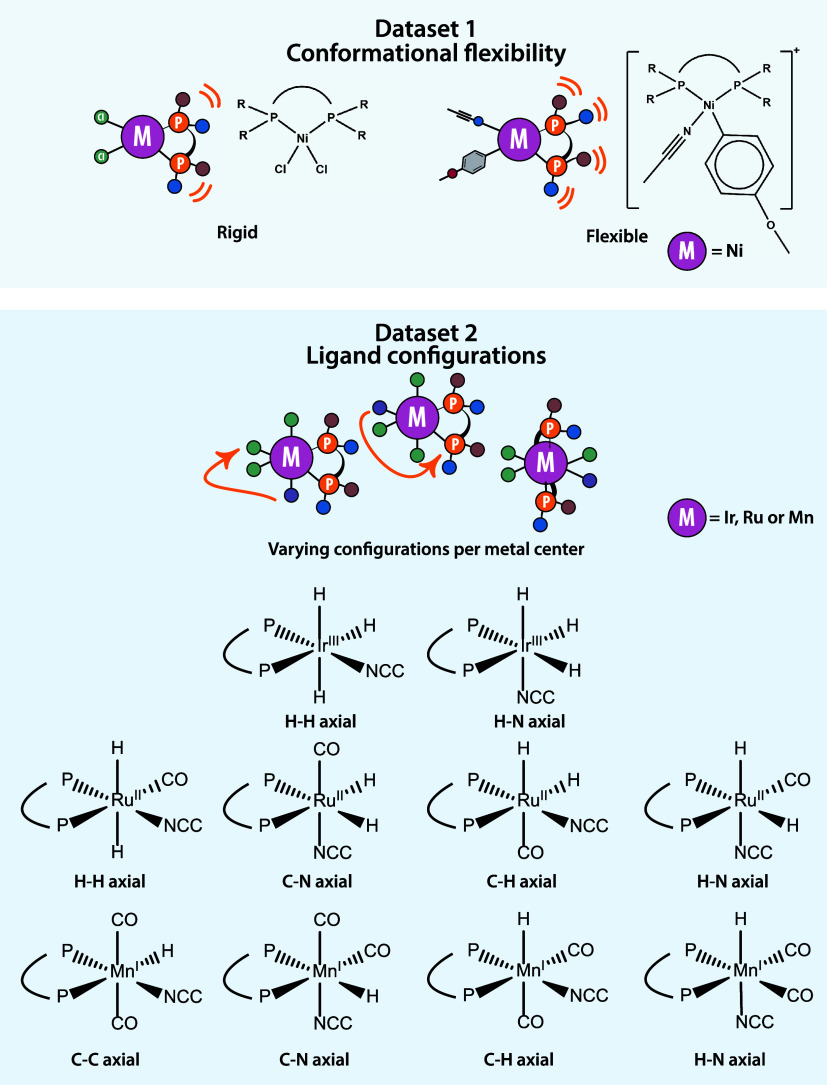
Overview of the two data sets used in this study. Top: Data set 1 contains conformers of square-planar Ni-based rigid and flexible model structures relevant to nitrile arylation, each with one of 25 bisphosphine ligands. Bottom: Data set 2 includes octahedral Ir, Ru, and Mn complexes relevant to hydrogenation chemistry, each with one of 88 bisphosphine ligands. Details on Data set 2 are available in our previous work.^22^ Figure adapted from ref 22 by A.V Kalikadien, N.J. van der Lem, C. Valsecchi, L. Lefort and E.A. Pidko. Available under a CC-BY 3.0 license. Copyright RSC.

Our case studies focus on accurately ranking the relative stabilities of either conformers (Data set 1) or ligand configurations (Data set 2). This ranking is performed by calculating the energy of the *i*-th conformer or configuration relative to a reference structure for the same ligand: Δ*E*_DFT/UMA_ = *E*_*i*_ – *E*_ref_. All relative energies are evaluated on DFT-optimized geometries to ensure consistency in structural input. To assess how well UMA reproduces DFT-based relative stabilities, conventional statistical metrics are employed, in particular, the Pearson correlation coefficient (*R* ^2^) and the root-mean-square error (RMSE). The strength of the linear correlation between DFT and UMA energies was quantified using Pearson’s correlation coefficient (*r*), and its square (*R* ^2^) is reported as a measure of how well the data follow a linear trend. with values closer to 1 indicating better agreement. The RMSE provides a direct measure of the average deviation between the UMA and DFT energies, expressed in kJ/mol.

## 3 Results and Discussion

Data set 1 consists of 23 ligands with 2100 conformers of the rigid dichloride model structure and 21 ligands with 3505 conformers of the flexible model structure. For comparative analysis, only ligands for which both the rigid and flexible model structures had fully converged conformer geometries were considered. This filtering resulted in 19 ligands, comprising 746 conformers for the rigid model structure and 1260 conformers for the flexible model structure. A comparison of relative energies computed by DFT and UMA on these structures (Figure 2) reveals excellent agreement, particularly for the rigid model’s conformers (*R* ^2^ = 0.96, RMSE = 2.4 kJ/mol), and reasonable correlation for the flexible model structures (*R* ^2^ = 0.68, RMSE = 9.1 kJ/mol). As expected, these results indicate that UMA has difficulties capturing energetic trends when the flexibility of complexes increase. While *R* ^2^ and RMSE capture the overall quality of the linear correlation and average energy deviation, they do not directly assess whether the relative ranking of conformers per ligand is preserved, which is an essential criterion for a reliable conformational analysis. Therefore, to more directly evaluate the ability of UMA to rank conformers correctly, we also computed Spearman’s rank-order correlation coefficient (ρ), a nonparametric metric that measures the monotonic relationship between two variables. In contrast to parametric measures such as Pearson’s correlation coefficient, which assumes linearity and normally distributed variables, Spearman’s rank-order correlation coefficient (ρ) operates solely on the ranked values. It, therefore, assesses whether two variables exhibit a monotonic relationship, regardless of the shape of their distributions. To ensure statistical robustness, we considered only ligands containing at least four conformer structures and required that the correlation be statistically significant (*p*-value <0.05). In addition, we adopted a threshold of ρ > 0.6 to define a “trusted” correlation, reflecting a moderate to strong monotonic agreement between UMA and DFT rankings. This cutoff is chosen to distinguish meaningful performance from weak or inconsistent ordering, as lower ρ values may indicate substantial deviations in the predicted energy ranking. By combining statistical significance with a minimum ranking strength criterion, this approach highlights cases where UMA is reliably predictive of the DFT-level ranking of relative stabilities.

**Figure 2:**
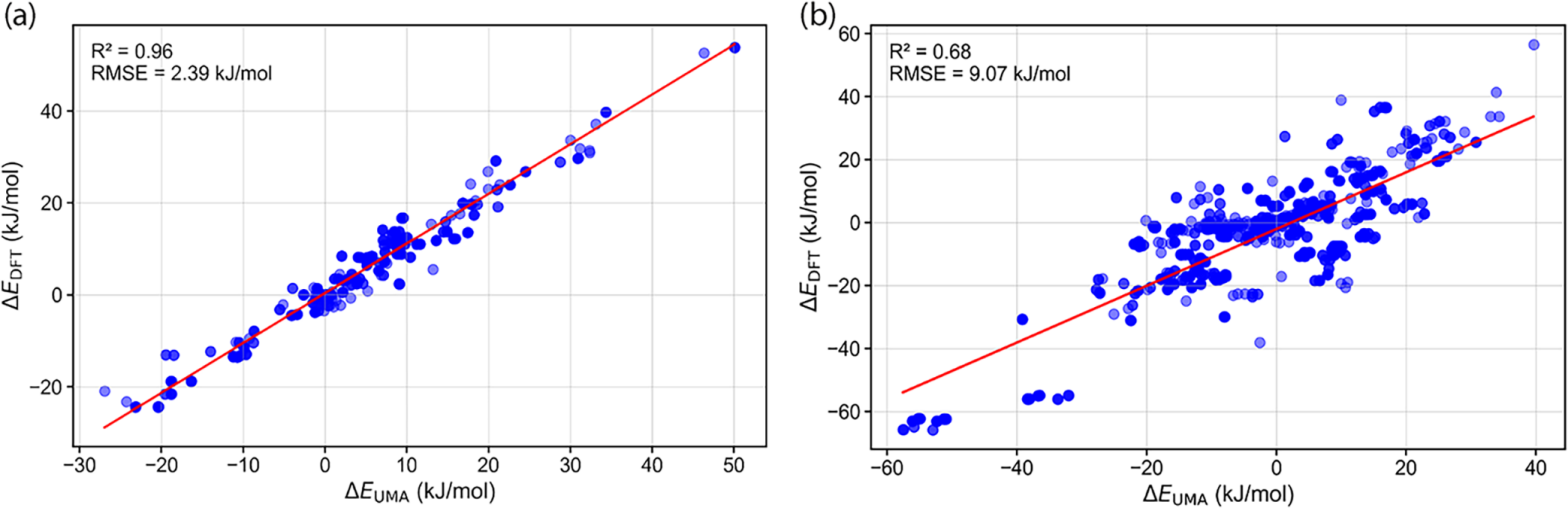
Correlation between DFT and UMA relative single-point energies for Data set 1 conformers: (a) rigid and (b) flexible model structures.

Our results show that for the rigid model structures, UMA reliably predicts the ranking for 84% of the ligands (Figure 3). However, this performance declines when applied to the flexible model structures, where 53% of the ligands exhibit a reliable ranking correlation. Notably, in two cases, a negative Spearman ρ was observed, indicating that UMA predicts the reverse energy ranking compared to DFT.

**Figure 3:**
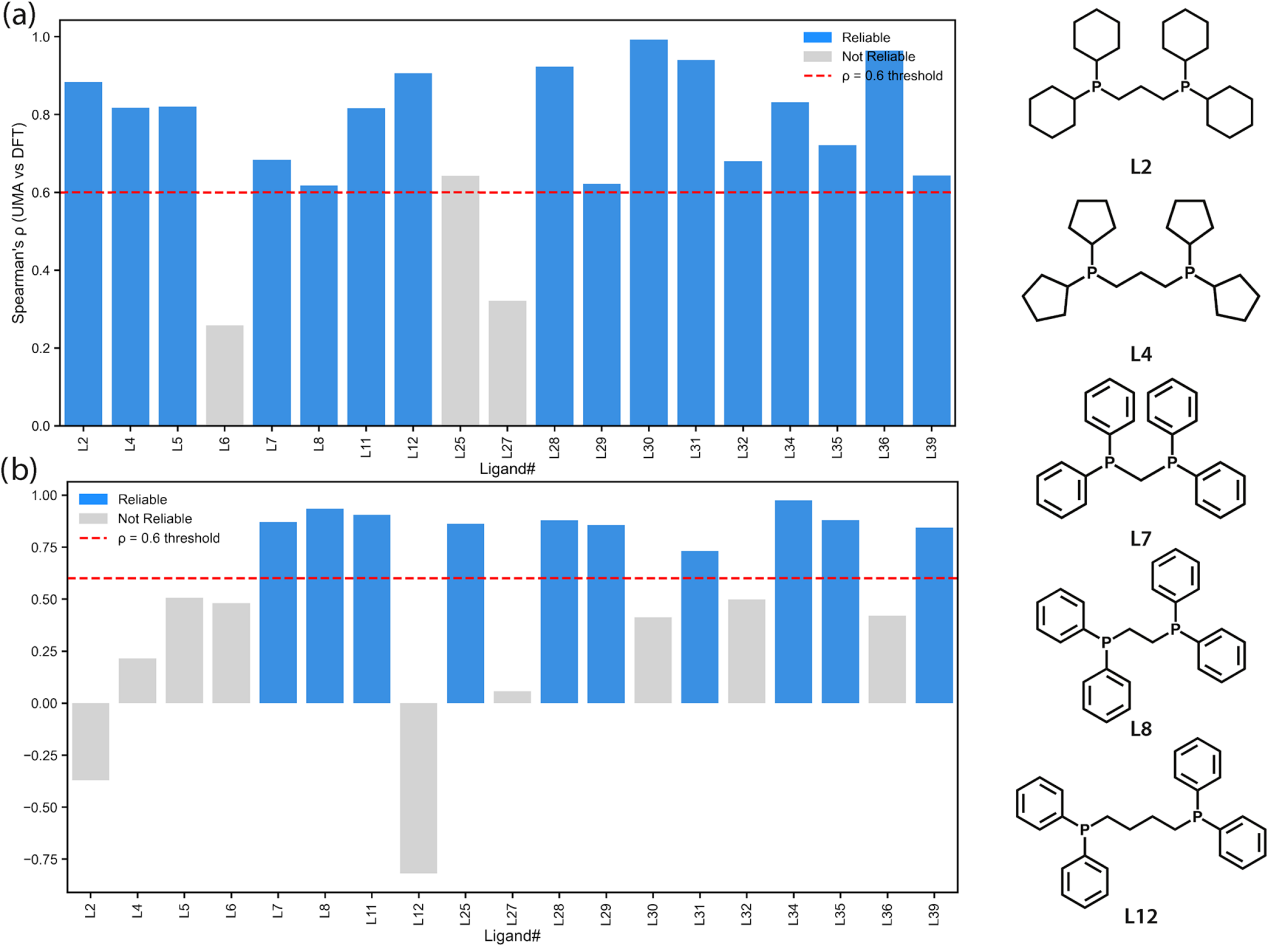
Spearman rank correlation (ρ) between UMA and DFT conformer energy rankings for Data set 1: (a) rigid and (b) flexible model structures. Bars are colored by reliability based on ρ > 0.6 (blue = reliable, *p* < 0.05 and ρ > 0.6; gray = not reliable). The red dashed line indicates the reliability threshold. The right-hand side shows representative bisphosphine ligands L2 (dCyhpp), L4 (Dcyppp), L7 (dppm), L8 (dppe), and L12 (dppb), illustrating differences in phosphorus substituents (phenyl, cyclohexyl, or pentyl) and linker length between donor atoms, which influence conformational flexibility and energy ranking performance.

To illustrate these trends, the right-hand side of Figure 3 presents five representative bisphosphine ligands (L2, L4, L7, L8, and L12), which differ primarily in the nature of the substituents on the phosphorus donors (phenyl, cyclohexyl, or pentyl) and in the length of the carbon linker between them. L2 (dCyhpp) and L4 (Dcyppp) are reliably predicted for the rigid model structure but not for the flexible model structure, with L2 even exhibiting a negative ρ. However, only eight conformers were identified for L2 in the flexible model structure with relative DFT energies differing by less than 2 kJ/mol. As expected, these are conditions under which UMA struggles to reproduce the DFT-level precision. L7 (dppm) and L8 (dppe), which have short linkers of one and two carbons, respectively, perform well for both rigid and flexible models, owing to their restricted conformational space. L12 (dppb) performs well in the rigid model structure but yields a negative ρ for the flexible model structure. In this case, only six conformers were found, again with relative DFT energies within 2 kJ/mol.

The tight energy ranges between conformers within Data set 1 provided a relatively strict test of UMA’s ranking capability, as energy differences between conformers are low and often approach chemical accuracy of the DFT method. To examine UMA’s performance under conditions where structural and energetic variations are greater, we next turned to Data set 2, which contains ligand configurations that span a much broader range of relative energies.

Data set 2 features 88 chiral bisphosphine ligands coordinated to various transition-metal complexes. After sampling stereoisomerism, 909 geometries were obtained. The metal centers, Ir­(III), Ru­(II), and Mn­(I), are stabilized with different auxiliary ligands, resulting in three distinct classes of complexes: [ligand]-IrH_3_(CH_3_CN), [ligand]-RuH_2_(CO)­(CH_3_CN), and [ligand]-MnH­(CO)_2_(CH_3_CN). A comparison of relative energies computed by DFT and UMA across this data set (Figure 4) again reveals excellent agreement (*R* ^2^ = 0.97, RMSE = 4.9 kJ/mol), highlighting UMA’s general applicability across multiple transition metals and ligand environments.

**Figure 4:**
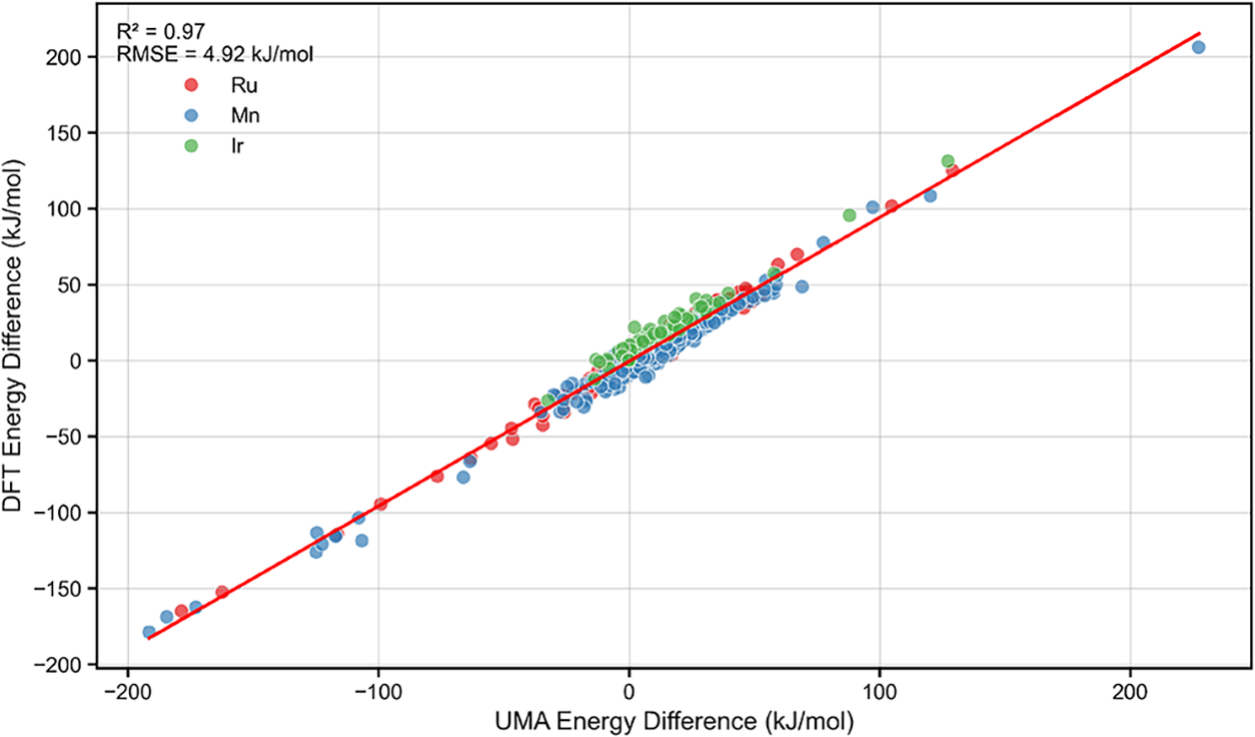
Correlation between UMA and DFT relative energies for Data Set 2 (configurations). Points are colored by metal center: Ir (green), Ru (red), and Mn (blue).

To more closely examine the reliability of energy rankings for individual ligands, we excluded the Ir­(III)-based complexes due to the presence of only two configurations per ligand, which is insufficient for rank correlation analysis. For the Mn­(I)- and Ru­(II)-based complexes, we again applied the significance and strength thresholds (*p*-value <0.05, ρ > 0.6) to define a “trusted” ranking. Based on this analysis, UMA is found to be reliably predictive for 53% of the ligand–metal combinations across the two metal centers (Figure 5). A closer examination per metal center reveals that 61% of the ligands are reliably predicted for Ru­(II)-based complexes, whereas this drops to 44% for Mn­(I)-based complexes. This difference is rooted in fundamental organometallic differences between the metal centers, where Mn­(I) complexes are known to exhibit more fluxional behavior. This can lead to a more complex and shallow potential energy surface, making the energy landscape harder to learn and predict reliably, especially with a general-purpose machine-learned potential, such as UMA. In contrast, Ru­(II) complexes tend to be more rigid and structurally well-defined under the same ligand field, contributing to the improved energy ranking accuracy observed for this class.

**Figure 5:**
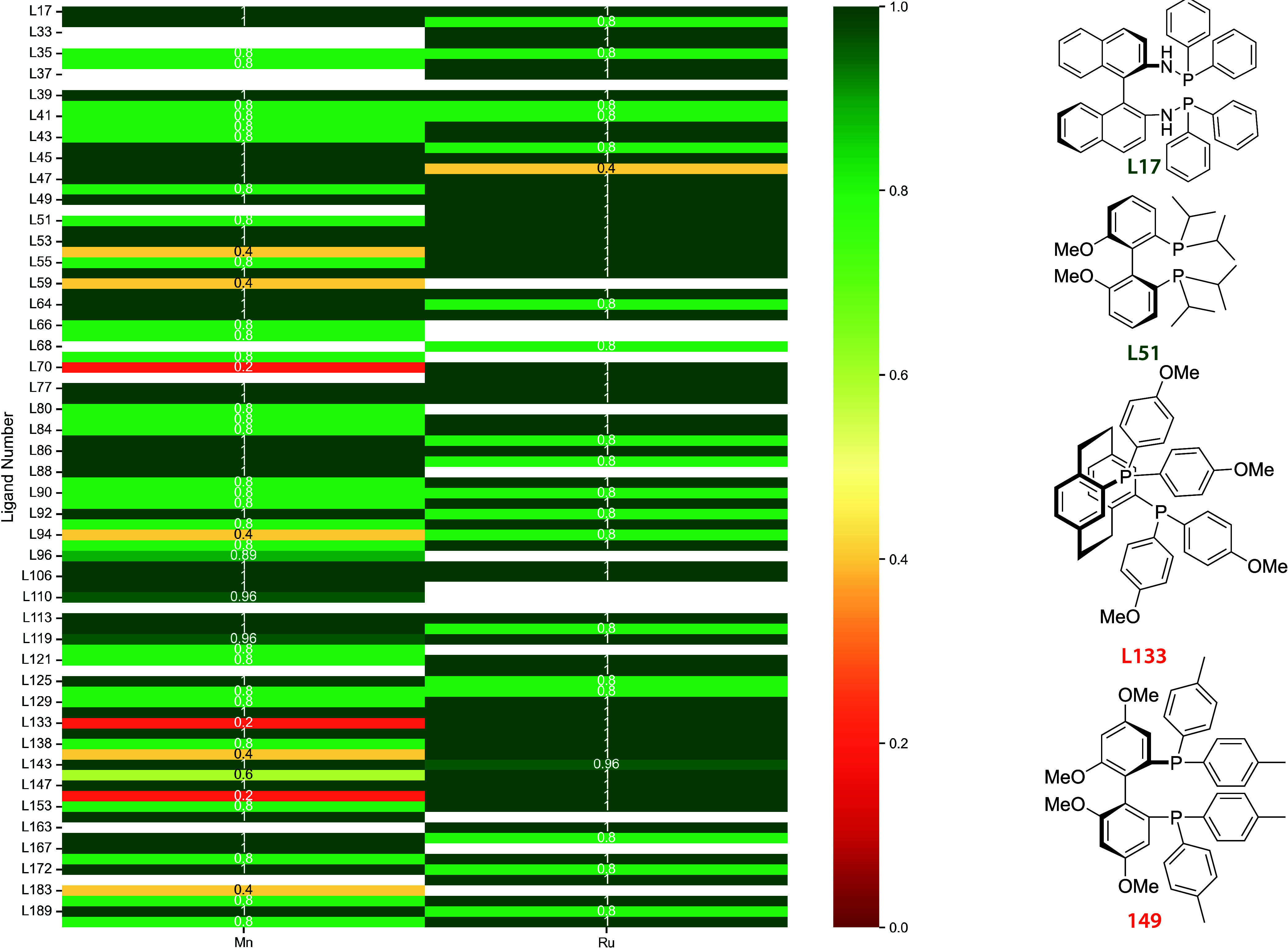
Spearman rank correlation (ρ) between UMA and DFT energy rankings for Data set 2: Mn­(I)- and Ru­(II)-based complexes. Cells are colored from red (low ρ) to green (high ρ). Ir­(III) complexes are excluded due to insufficient configurations for ranking. The right panels show representative chiral bisphosphine ligands: L17 ((R)-BINAM-P) and L51 ((S)-iPr-BIPHEP) (green) achieve perfect agreement for both metals, while L133 ((R)-An-PhanePhos) and L149 ((R)-Tol-GarPhos) (orange) are reliable for Ru­(II) but not for Mn­(I).

The right-hand side of Figure 5 highlights four representative bisphosphine ligands that exemplify these trends. L17 ((R)-BINAM-P) and L51 ((S)-iPr-BIPHEP) are colored green because they achieve a near-perfect ρ of 1.0 for both Mn­(I) and Ru­(II) complexes. In both cases, the relative energies between configurations are substantial, with L17 showing differences of at least 6 kJ/mol (up to 43 kJ/mol for the H–H configuration and 34 kJ/mol for the C–N configuration in Ru complexes) and L51 showing differences of at least 4 kJ/mol (C–N configuration in Mn) and typically exceeding 15 kJ/mol for both metals. These large energy differences appear to be easily predicted by UMA.

In contrast, L133 ((R)-An-PhanePhos) and L149 ((R)-Tol-GarPhos) illustrate cases in which UMA performs well for Ru­(II) complexes but fails for Mn­(I). For L133, Ru complexes display relative energy differences of at least 6 kJ/mol, whereas Mn complexes that feature C–C and C–H configurations differ by less than 2 kJ/mol from the reference. This aligns with our earlier observation that these Mn complexes can exhibit substantial structural isomerism, presenting multiple ligand configurations within 10 kJ/mol.^22^ For L149, the Ru C–N configuration is separated from the reference by only 2.3 kJ/mol, yet is still ranked correctly, while the Mn C–C configuration differs by just 0.2 kJ/mol and the remaining Mn configurations lie within 5 kJ/mol, leading to inaccurate rankings. However, these examples reinforce the observation that only small energy differences that are near the DFT accuracy pose a challenge for UMA.

## 4 Conclusions

Our results demonstrate that UMA represents a significant step forward in the development of MLIPs. With near-DFT accuracy in energy prediction across a wide range of ligand conformers and configurations, UMA enables a rapid and scalable analysis in computational chemistry. For example, single-point energy evaluations using UMA can be performed in seconds on consumer-grade GPUs, in stark contrast with the CPU time required for equivalent DFT calculations. This speedup offers clear advantages in high-throughput screening and early stage catalyst design workflows. However, the current generation of MLIPs, including UMA, is not without limitations. While they are highly effective at predicting relative energies and forces, they do not yet provide access to electronic structure information such as the electron density, which remains essential for understanding charge distribution, reactivity, and spectroscopic properties. In addition, UMA is trained exclusively on gas-phase data and does not currently incorporate solvation effects. Because the solvent can substantially influence conformational energetics and catalytic behavior, extending UMA with implicit- or data-driven solvent models represents an important direction for future development. Such advances would broaden the applicability of MLIPs to solution-phase catalysis and other realistic chemical environments.

In Data set 1, reliable rankings are achieved for 84% of ligands in the rigid [ligand]-Ni­(II)-Cl_2_ model structures, but this drops to only 53% for the more flexible [ligand]-Ni­(II)-(CH_3_CN)­(−pOMe­(C_6_H_4_)) model structures, where asymmetric coordination and increased conformational freedom pose additional challenges. Ligands such as L7 (dppm) and L8 (dppe) are well-predicted in both rigid and flexible cases due to their short linkers and restricted conformational space, whereas L2 (dCyhpp) and L12 (dppb) are ranked less accurately for the flexible model structures, often when relative DFT energies between conformers differ by less than 2 kJ/mol. This is a regime where errors in ranking relative stabilities may have limited impact on equilibrium ground-state populations, and similar differences in ranking could appear from any DFT methodology by changing basis sets or functionals within the same rung on Jacob’s ladder.

A similar pattern is observed in Data set 2, where UMA reliably ranks configurations for 61% of Ru­(II)-based and 44% of Mn­(I)-based complexes. Ligands with large relative configuration energy gaps, such as L17 ((R)-BINAM-P) and L51 ((S)-iPr-BIPHEP), achieve near-perfect correlations for both metals, while ligands with small energy separations within the error range of the DFT perform worse. These findings underscore that UMA as a general MLIP can be a powerful tool to achieve near-DFT accuracy for both rigid and structurally well-defined systems as well as fluxional and highly flexible complexes.

Finally, it is important to acknowledge the paradigm shift that MLIPs introduce. Although DFT itself is an approximation, the behavior and limitations of exchange-correlation functionals, basis sets, and dispersion corrections have been extensively studied and understood over decades. These methods are rooted in physics. In contrast, general-purpose ML models such as UMA are trained as black boxes on vast data sets, and their performance is not easily interpretable. This abstraction risks concealing underlying failures if used uncritically. As the field transitions into a post-UMA era, it will be crucial to combine the speed of ML with validation and domain expertise to ensure robust and meaningful chemical insights. Importantly, the instances where UMA fails to reproduce DFT rankings occur predominantly in near-degenerate energy regimes, where DFT itself cannot provide a uniquely reliable ranking. This exemplifies that expert judgment remains essential when interpreting such cases, even as MLIPs like UMA enable rapid and accurate exploration of broader regions of chemical space.

## Data Availability

The data sets, an overview of ligands and the code for reproducing the analysis presented in this study are available with an extensive readme via 4TU.ResearchData at 10.4121/6b178daf-e1c0-4c99-840f-06d382f37945.
